# A Systematic Literature Review and Bibliometric Analysis of Workplace Coaching

**DOI:** 10.12688/f1000research.171808.2

**Published:** 2026-04-29

**Authors:** Itam Urmila Jagadeeswari, Nidhi Shukla, Mercy Toni, Keerti Mishra

**Affiliations:** 1 Department of Commerce, Manipal Academy of Higher Education, Manipal, Karnataka, India; 2 Presidency Business School, Presidency College, Bengaluru, Karnataka, India; 3 Department of Economics and Finance, University of Nizwa, Nizwa, Ad Dakhiliyah ‍Governorate, Oman; 4 Sushant School of Art and Architecture, Sushant University, Gurugram, Haryana, India

**Keywords:** Workplace Coaching, Leadership Coaching, Executive Coaching, Employee Coaching, Systematic Literature Review.

## Abstract

Workplace Coaching has evolved significantly over the last two decades and has become a mainstream global activity in business organizations. The concept has been recognized for its potential in enhancing the well-being and performance of individuals, groups, and leaders, making it a popular intervention in modern organizations. Therefore, this study provides a comprehensive understanding of workplace coaching research from 2000 to 2025 by examining global trends in terms of publications, contributors, keyword co-occurrences, and thematic clusters, utilizing a systematic literature review with bibliometric analysis. The review was conducted to synthesise the available literature on workplace coaching to suggest future trends. The Scopus and Web of Science databases were used to explore and analyze published works using the PRISMA framework and VOSviewer software. A total of 343 published journal articles were considered in the analysis, eliminating duplication and minimizing potential risks during the screening process. The findings reveal a significant growth in academic interest in coaching in workplace research, with four key thematic clusters emerging: Coaching for Workplace Learning and Development, Multifaceted advantages of Executive and Leadership Coaching, Dimensions of Workplace Coaching, and Employee Coaching for Human Resource Management and Development. The study findings serve as a foundation for future studies that explore under-researched areas, such as the application of artificial intelligence in coaching practices, ethical considerations, digital transformation, sustainability in leadership, and employee well-being practices. Moreover, this study reveals the potential of meta-analyses and systematic reviews that incorporate grey literature to provide a more comprehensive understanding of coaching effectiveness. Comparative studies across different sectors, countries, and regions can help consolidate the existing findings and uncover new insights. These limitations and opportunities present avenues for future research, motivating researchers to advance and refine existing literature.

## 1. Introduction

Coaching, whether it is leadership, executive, or employee coaching, is a widely recognised tool for enhancing performance, developing skills, and fostering personal and professional growth within organisations. Leadership coaching focuses on developing leadership skills, enhancing the leader's ability to support their team, and promoting constructive leadership behaviors, such as delegation and individualized consideration ( Anthony, 2017; Brennan, 2012; Weihrauch et al., 2022). Executive coaching aims to help executives navigate organizational changes, improve their psychological and behavioral skills, and achieve work-related goals. This is associated with increased goal attainment, resilience, and leadership self-efficacy ( Anthony, 2017; Grant, 2014; Nicolau et al., 2023). Employee coaching enhances employee performance, motivation, and loyalty, helping employees solve their own problems and improve their thinking and performance ( McGuffin & Obonyo, 2010).

The concepts of leadership, executive, and employee coaching are widely recognized as effective tools for leadership and executive development, designed to enhance the skills, behaviors, and overall effectiveness of employees within an organization. Leadership and executive coaching have evolved from contrasting traditions, with leadership coaching shifting towards a collective phenomenon known as leadership-as-practice (L-A-P) ( Ratiu et al., 2017). Executive coaching emerged from management changes in the for-profit sector during the closing decades of the twentieth century, initially focusing on senior management and later expanding to include younger, high-potential employees and middle managers. Leadership coaching emphasizes a collective approach, while executive coaching is geared towards supporting leaders at all levels in driving organizational change ( Bennett & Bush, 2013). Employee coaching, often referred to as managerial coaching, focuses on facilitating employees’ learning process to enhance their performance and effectiveness in organizations ( Ratiu et al., 2017).

The focus of leadership coaching has evolved significantly over time to address a diverse range of challenges in the workplace. Initially, in the 1990s, leadership coaching primarily concentrated on performance management with structured, step-by-step training programs aimed at enhancing performance ( Grant, 2014). The second generation (2000s) continued performance focus but introduced proprietary “leader as coach” programs. These programs were still mechanistic and did not fully address the complexities of modern organizational challenges ( Grant, 2014). The third generation is contemporary, emphasizing both performance and well-being, with a focus on sustainable and personally meaningful development. Encourages quality conversations and a developmental approach to creating a supportive culture ( Bennett & Bush, 2013). However, as organizational contexts became more uncertain and rapidly changing, this mechanistic approach proved insufficient.

Furthermore, executive and leadership coaching are increasingly recognized as effective strategies for enhancing employee well-being and organizational performance. Research in this direction has explored coaching and its impact on employees, focusing on their psychological well-being, engagement, and job satisfaction. Coaching leadership has both positive direct and indirect effects on employee well-being, knowledge-sharing behavior, and innovative employee behavior ( Tanuwijaya et al., 2025). It is positively related to work engagement through the mediation of psychological capital and in- and extra-role performance, as well as work engagement ( Peláez Zuberbuhler et al., 2020). Kim & Park (2023) found that leadership coaching has a positive effect on psychcap and organizational commitment, which in turn positively affects organizational effectiveness. Coaching leadership can help decrease burnout symptoms and increase vigor in leaders, thereby enhancing their overall well-being. Romão et al. (2022) demonstrated a negative impact on employees’ turnover intention and a positive impact on their happiness, with happiness mediating the relationship between coaching leadership and turnover intention.

Despite the popularity and usage of these concepts, the mechanisms through which they facilitate development and the factors contributing to their success remain under-researched. To address this gap, systematic literature reviews (SLR) in this domain are considered important, and the literature also shows a scarcity of such review articles. SLRs help evaluate empirical evidence for the effectiveness of coaching interventions. They provide a comprehensive overview of how coaching impacts productivity and performance in organizations ( Grover & Furnham, 2016; Plotkina & Sri Ramalu, 2024). Understanding the mechanisms underlying coaching is crucial. SLRs can identify factors, such as coach characteristics, coachee aspects, and the coach-coachee relationship, that contribute to successful coaching outcomes ( Athanasopoulou & Dopson, 2018; Lai & Palmer, 2019). Although most SLR papers published in the domain are of a qualitative nature, bibliometric studies or meta-analysis research lay a foundation for key research areas, themes, and frameworks ( Kakkar & Sachdeva, 2025).

Tracing the journey of this evolving field, this study presents a bibliometric analysis of 343 articles published from 2000 to 2025, drawing on verified data from Scopus and the Web of Science. It examines publication trends, geographic reach, citation patterns, and author contributions, including institutional affiliation. By mapping co-authorship networks and keyword connections, this study uncovered key research hubs, emerging topics, and global collaboration patterns, providing fresh insights into the field’s growth and future trajectory. To guide this study, the following research questions were formulated to provide a detailed exploration of key aspects within the field.

*RQ1: How has the research on workplace coaching progressively developed over time?*

*RQ2: What are the most significant contributors (authors, articles, institutions, countries, and publications) that have shaped the development of workplace coaching?*

*RQ3: How can network diagrams and cluster visualizations be constructed to illustrate the relationships and connections among different authors and keywords?*

*RQ4: What are the key research gaps that can pave the way for future studies in workplace coaching?*

This research article is divided into six sections: the first section provides the introduction and outlines the research idea. The second section highlights the existing literature, theories, and frameworks in this area. Section three provides a detailed explanation of the methodology used in this study. The fourth section described the bibliometric findings of the research, followed by the fifth section, which highlights the important discussions and implications of the study. The last section includes the conclusions, limitations, and future research directions.

## 2 Literature review

### 2.1 Leadership coaching, executive coaching, and employee coaching

Leadership coaching is a tool for leadership development that involves a structured process to enhance leaders’ skills and effectiveness. This requires coaches to have both methodological and leadership knowledge to address specific framework conditions and requirements ( Weihrauch et al., 2022). It is used to build leadership capacity in individuals and institutions by enhancing professional relationships and maximizing the potential to meet personal and professional goals ( Robertson, 2008). Leadership coaching is also seen as a way to manage employees by focusing on their well-being and business success ( Mollaret & Claudepierre, 2016).

Employee coaching is a facilitating relationship between managers and their subordinates aimed at improving individual or organizational performance. This is based on the premise that employees are capable of improving and maintaining good relationships with their managers ( Koskinen & Anderson, 2023). This type of coaching is increasingly expected of managers and focuses on building relationships based on compassion, mutual trust, respect, and collaboration. It is used to help employees improve performance, communication, and cooperation within the organization ( Dromantaitė, 2021).

Executive coaching is a process designed to help professionals, particularly leaders and top management, reach organizational goals by learning new competencies and implementing behavioral changes ( Brunning, 2018; Milaré & Yoshida, 2009). It is a popular leadership development intervention that helps executives become independent learners and coach themselves and others. Executive coaching is also seen as a higher-grade coaching intervention that focuses on systemic-psychodynamic theory within an organizational context ( Rajasinghe & Garvey, 2023). It aims to inspire leaders to improve organizational performance and navigate challenges, such as technological impacts and increasing competition ( Milaré & Yoshida, 2009; Rajasinghe & Garvey, 2023).

### 2.2 Differences and uniqueness of leadership, executive, and employee coaching

The literature on coaching within organizational contexts reveals significant overlaps and distinctions among employee coaching, leadership coaching, and executive coaching. All three types of coaching aimed to enhance individual performance and development, as given in Table 1. Employee coaching focuses on improving specific job-related skills and performance ( Hamlin et al., 2006; Joo et al., 2012; Ratiu et al., 2017). Leadership and executive coaching often focus on developing broader leadership competencies and strategic thinking ( Albizu et al., 2019; Brunning, 2018; de Villiers & Botes, 2013; Williams & Lowman, 2018).

**Table 1.** Definitions and evolution of executive, leadership, and employee coaching.

Type of coaching	2010-2015 definition	2016-2025 definition	Focus	Applications
Executive Coaching	Process to reach organizational goals by learning new competencies ( Milaré & Yoshida, 2009)	A process where cognition, attitudes, and emotions bring about change ( Bakhshandeh et al., 2023)	Goal-oriented, systemic-psychodynamic theory ( Brunning, 2018)	Behavioral changes, organizational performance ( Tǎnǎsescu et al., 2009)
Leadership Coaching	Dynamic relationship for leadership development ( MacKie, 2015)	Critical approach for developing effective leadership ( Basher, 2025)	Client-coach relationship, leadership behaviors ( Ely et al., 2010)	Continuous development, managing transitions ( Alias et al., 2020)
Employee Coaching	Facilitating relationships to increase performance ( Gregory & Levy, 2010)	Enhancing performance and well-being sustainably ( Grant, 2017)	Supervisor-subordinate relationship ( Dromantaitė, 2021)	Performance improvement, communication, and cooperation ( Bekmezci et al., 2025)

Note - This table summarizes key definitions, focus areas, and practical applications of executive, leadership, and employee coaching from 2010–2025. It highlights the evolving perspectives and approaches in each coaching type across two periods, providing a comparative view for scholarly and organizational contexts.

Both leadership and executive coaching have been shown to positively impact organizational performance and employee development. Research indicates that coaching can increase leaders’ commitment and self-efficacy, leading to improved organizational performance ( Alves & Figueiredo, 2024; Baron & Morin, 2010; Williams & Lowman, 2018). Coaching has been found to be effective in promoting individual-level learning in an organizational setting, with significant positive effects on outcomes such as self-efficacy, psychological capital, and resilience ( Gladis, 2007; Nicolau et al., 2023). which is crucial for maximizing their impact and value. This involves managing coaching initiatives, including executive coaching, internal coaching, coaching by managers, and peer coaching, to ensure their effectiveness ( Hunt & Weintraub, 2007). Organizations should consider coaching from a systemic perspective, taking into account individual and situational variables, and implement programs with multiple sessions spread over several months to enhance return on investment ( Baron & Morin, 2010).

Studies have found that one of the challenges of integrating coaching in organizational settings is the need to formalize coaching processes and structures, particularly for internal coaching. While many individuals within organizations already engage in informal coaching, there is a need to establish formalized coaching cultures to promote dialogue and feedback between leaders and teams at all levels of the organization, and there ( Abel & Nair, 2016; Riddle, 2016). There is a lack of research-informed evidence to overcome the challenges faced by organizations when employing external coaches, such as what selection criteria or evaluation benchmarks to use ( Lai & Palmer, 2019).

Therefore, leadership and executive coaching play crucial roles in driving organizational change and developing leadership competencies. Both types of coaching have been shown to positively impact organizational performance and employee development. Implementing coaching programs strategically and formalizing coaching processes and structures are essential for maximizing the effectiveness of coaching initiatives in organizational settings. However, there is a need for further research to provide evidence-based guidelines for employing external coaches and establishing formalized coaching cultures within organizations.

### 2.3 Theoretical foundation of executive, leadership, and employee coaching

The theoretical foundations of executive, leadership, and employee coaching in the literature are supported by various key concepts and developments ( Table 2). The Control Theory Framework (CTF) emphasizes the importance of goals and feedback in executive coaching. This suggests that coaches help executives become effective self-regulators by engaging in goal setting and feedback-seeking ( Gregory et al., 2011). Action Frame Theory (AFT), developed from social action and functional job analysis theories, provides a structured approach to integrate executive and system foci during coaching engagements ( Cocivera & Cronshaw, 2004). Executive coaching often applies systemic-psychodynamic theory in the context of organizational life. Focusing on goal-oriented coaching within a managerial/leadership context ( Brunning, 2018). Formulation-Based Approach (FBA), rooted in applied psychology, helps coaches navigate the complexities of their practice by systematically understanding the context of the coachee ( Kovacs & Corrie, 2021). Ecosystems Theory (ET) involves understanding the coachee’s context at multiple levels (organizational, team, intrapersonal) to design effective coaching interventions ( Bhatnagar, 2021). Psychodynamic, cognitive-behavioral, and systems approaches have been developed in the field of psychology to address various challenging problems in executive coaching. This multifaceted approach involves interventions addressing individual, group, and organizational-level processes ( Turner & Goodrich, 2010).

**Table 2.** Summary of theoretical foundations, key concepts, empirical evidence, and gaps.

Parameters	Executive coaching	Leadership coaching	Employee coaching
**Theoretical Frameworks**	Systemic-psychodynamic theory - Cognitive-behavioral approaches - Solution-focused coaching - Transformative learning theory	Transformational leadership model - Emotional and behavioral response frameworks	Humanistic coaching integrated with self-determination theory - Cognitive-behavioral coaching
**Key Concepts**	Managerial flexibility- Quality integrated thinking, confidentiality, trust- Addressing dysfunctional belief systems	Assertive communication skills- Motivation of subordinates- Emotional intelligence	Autonomy, competence, relatedness - Learning and development processes
**Empirical/Quantitative Evidence**	Positive impact on managerial flexibility - Importance of coach quality and coachee readiness	Increased leadership behavior scores - Influence on employee attitudes and behaviors	Enhanced employee outcomes through psychological needs fulfillment
**Practical Implications**	Development of executive coaching programs - Multifaceted interventions for leadership issues	Coaching programs for mid-level managers - Frameworks for values-based leadership	PARR framework for employee development- Integrative coaching models
**Research Gaps**	Need for more empirical research on coaching processes - Exploration of transformative learning elements	Cross-cultural comparisons of coaching effectiveness	Lack of theory on coaching behaviors on outcomes

Note - This table outlines the comparative theoretical foundations, salient concepts, supporting evidence, practical implications, and research gaps for executive, leadership, and employee coaching. It offers a synthesized view to facilitate understanding of coaching dimensions and highlight areas for future research in organizational contexts.

Leadership coaching involves building a coaching relationship within the leader-follower dyad, where the leader supports the follower’s personal development while directing them toward common goals and managerial tasks ( Cannata & Danels, 2024). General Systems Theory (GST) and cybernetics help leaders develop holistic problem-solving strategies, improve communication, and foster resilience and adaptability ( Harris, 2024). The Leader-Culture Fit Framework (LCFF) was developed based on person-environment fit theory and aligns leader development strategies with organizational culture to support positive culture change ( Huffington, 2018). The Transformational Leadership Model (TLM) evaluates management behaviors and emphasizes the development of managerial coaching skills, assertive communication, and the motivation of subordinates ( Ratiu et al., 2017). The contextualized Approach (CA) considers the organizational context conveyed through the coachee’s interactions, integrating person-role-system models ( Nieminen et al., 2013).

The temporal map of coaching framework links seminal concepts in psychology to the coaching process, focusing on different stages of change (awareness, willingness, goal setting, and reflection) to enhance employee well-being and functioning ( Theeboom et al., 2017). Social Exchange Theory (SET) and Resource-Based View theories (RBVT) explain how coaching impacts individual and organizational performance, emphasizing the importance of social interactions and resource management ( Utrilla et al., 2015). The Co-Route to Coaching Partnership (CPM) model emphasizes the importance of compassion, mutual trust, respect, and collaboration in effective employee coaching relationships ( Koskinen & Anderson, 2023). These frameworks offer a structured approach to understanding and implementing coaching practices across various contexts, ensuring that coaching interventions are both effective and aligned with organizational objectives.

## 3. Research methodology

The current study employs a combination of bibliometric analysis and network analysis to illustrate the overarching trends in executive and leadership coaching over time. This is based on scientometrics, which focuses on the quantitative examination of scientific literature to identify patterns and trends within the research field ( Hood & Wilson, 2001). It draws on a foundational concept, such as Bradford’s Law, which examines the distribution of articles across journals ( Peritz, 1990); Lotka’s Law, which outlines patterns in author productivity ( Bacaër, 2011); and Zipf’s Law, which aids in analyzing the frequency of keywords, thus revealing thematic trends ( Zipf, 1950). Additionally, it incorporates Citation Analysis Theory to assess influential works ( Garfield, 1955). Network Theory explores relationships among research entities ( Newman et al., 2021) and knowledge domain visualization maps thematic clusters and trends. Bibliometric analysis was used to examine the effectiveness and influence of various research topics and themes within a field of study, as well as to illustrate the relationship between the various themes and topics ( Cobo et al., 2011). Effectively conducted bibliometric studies provide a robust basis for advancing a field in novel and innovative ways, in terms of providing a comprehensive understanding, identifying gaps in existing knowledge of research, generating innovative research ideas, and wisely offering their findings within the scholarly landscape ( Donthu et al., 2020).

### 3.1 Database selection, keyword search parameters, and data screening

Researchers have successfully utilized SCOPUS and Web of Science (WoS) to implement bibliometric analysis. The data were collected between 2000 and 2025 using two thoroughly considered data repositories to conduct a systematic literature review analysis ( Kakkar & Sachdeva, 2025). The SCOPUS database is considered to be one of the largest databases in terms of the number of journals and publisher categories. Similarly, WoS also holds high-quality, impactful journals that are equally suitable for bibliometric analysis ( Donthu et al., 2020; Lim et al., 2024). Both databases are considered multidisciplinary in nature, and together they have become the largest collection of documents and biographical information. These two databases have systematized databases and dominate cross-disciplinary datastores, where editorial teams ensure peer-reviewed publications within social sciences research ( Goyal & Kumar, 2021). Open-access databases, such as Google Scholar, Dimensions, and PubMed, may encompass a wide range of publications; however, they may not be peer-reviewed or managed by editorial boards, which can lead to greater inaccuracy and lower reliability.

**Figure 1.**
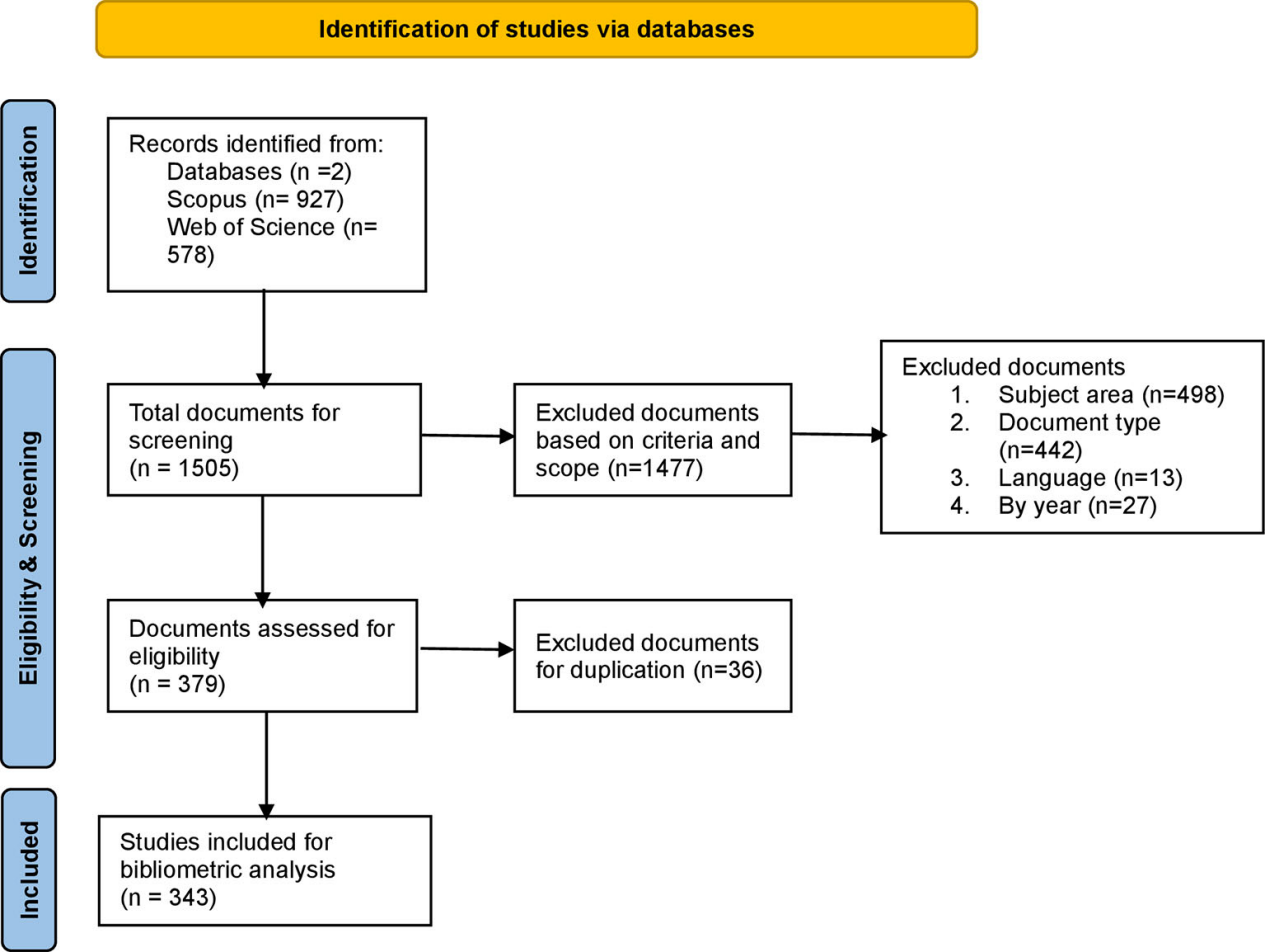
PRISMA methodology for identification, screening, and inclusion of documents. This figure illustrates the PRISMA flow diagram depicting the systematic process of identification, screening, eligibility assessment, and inclusion of studies from SCOPUS and Web of Science databases for bibliometric analysis of workplace coaching literature.

First, the keyword search needs clarity on certain keywords that can be identified from the existing literature review. The authors employed executive, leadership, or employee coaching, utilizing the Boolean operator “OR” to facilitate the extraction of data from both the SCOPUS and WoS databases. The search string was formulated using the latest foundational works that specifically examined coaching in the workplace context, rather than general coaching ( Kakkar & Sachdeva, 2025). Authors used PRISMA (Preferred Reporting Items for Systematic Reviews and Meta-Analyses), including 27 checklist, as a protocol for conducting a systematic literature review, especially in management research ( Haddaway et al., 2022). The inclusion criteria used to acquire the right research articles from both the databases include – timeframe – 2000 to May 2025; documents published in English, review and research articles published in journals, indexed by SCOPUS (Q1 & Q2) and WoS (ESCI and SSCI indexing), and further details related to subject areas are shown in Figure 1 and Table 3.

**Table 3.** Summary of search strategy using PRISMA guidelines.

Type	Criteria
Database	Scopus and Web of Science
Search string	“Workplace Coaching” OR “Coaching at Workplace” OR “Leadership Coaching” OR “Executive Coaching” OR “Employee Coaching”
Period	2000 to 2023
Subject area	Business Management
Document Type	Article
Source of the publications	Journal
Language	English
Search date	02-Jun-25

Note - This table presents an overview of the systematic search strategy applied in accordance with PRISMA guidelines, detailing key database sources, search terms, timeframe, inclusion criteria, and language settings for identifying relevant peer-reviewed literature on workplace and coaching practices.

Initially, 927 and 574 research articles were explored from the SCOPUS and WoS databases, respectively. However, in later stages, considering the inclusion and exclusion criteria of the search, a total of 343 efficient articles were used for the scientometric analysis using Vos Viewers and R-studio. Different types of software are available for conducting bibliometric analysis, such as CiteSpace, Gephi, BiblioShiny, and Vosviewer; however, this research utilized a free, open-source viewer for analysis. It is used to construct and visualize bibliometric networks from almost all databases, such as SCOPUS, WOS, Dimensions, Google Scholar, and PubMed. Vosviewer software is known for its user-friendly interface and ability to generate informative visualization structures ( Kakkar & Sachdeva, 2025).

To enhance reproducibility and methodological transparency, the complete database-specific search strings used for both Scopus and Web of Science are reported verbatim below.

Scopus search string: TITLE-ABS-KEY ((“executive coaching” OR “leadership coaching” OR “employee coaching” OR “workplace coaching”)) AND PUBYEAR > 1999 AND PUBYEAR < 2026 AND ( LIMIT-TO ( DOCTYPE, “ar”) OR LIMIT-TO (DOCTYPE, “re”)) AND (LIMIT-TO (LANGUAGE, “English”)).

Web of Science search string: TS = (“executive coaching” OR “leadership coaching” OR “employee coaching” OR “workplace coaching”) AND PY = (2000-2025) AND DT = (Article OR Review) AND LA = (English) AND SU = (ESCI OR SSCI). Both strings employed Boolean operator OR with field tags, language filters (English), document-type filters (journal articles and review articles), and a timeframe of 2000 to May 2025.

### 3.2 Bibliometric analysis parameters and thresholds

VOSviewer (version 1.6.20) was employed for co-authorship, keyword co-occurrence, and citation analyses. For co-authorship analysis, the minimum number of documents per author was set to 2, and the minimum number of citations was set to 5, yielding a network of the most productive and cited contributors. For keyword co-occurrence analysis, author keywords were selected with a minimum occurrence threshold of 3, producing a network of 56 keywords that met the criteria out of 801 total keywords. The association strength normalization method was applied to account for differences in keyword frequency. For citation analysis, a minimum citation count of 10 per document was required. The counting method was set to “full counting” to ensure equitable attribution across all co-authors. All visualizations were generated using the default VOSviewer settings for cluster resolution (1.0) and the number of iterations (1000), ensuring replicable and stable network structures.

## 4. Data analysis and findings

This section analyzes the results obtained from the SCOPUS and WoS databases regarding the annual scientific outcomes, including the most relevant sources, most influential authors and works, affiliations, documents, citations, co-occurrences, countries, and important elements that require further consideration. Table 4 summarizes the workplace coaching data considered in this study, drawn from both databases.

**Table 4.** Data summary from scopus and Web of Science (WoS).

Description	Results
Primary focus on data	SCOPUS and WoS
✓ Time period	2000:2025
✓ Sources	Journals
✓ Documents	343
✓ Annual growth rate	13.92
✓ Document average age	3.998
✓ Avg citations per document	21.25
Documents Contents	
✓ All Keywords	867
✓ Author Keywords	790
✓ Index Keywords	115
✓ Total number of authors	527

Note - This table provides a summary of descriptive data characteristics and document metrics from SCOPUS and Web of Science for the period 2000–2025, including source details, document counts, growth rates, citation averages, and keyword statistics to describe the scope and content of the dataset.

**Figure 2.**
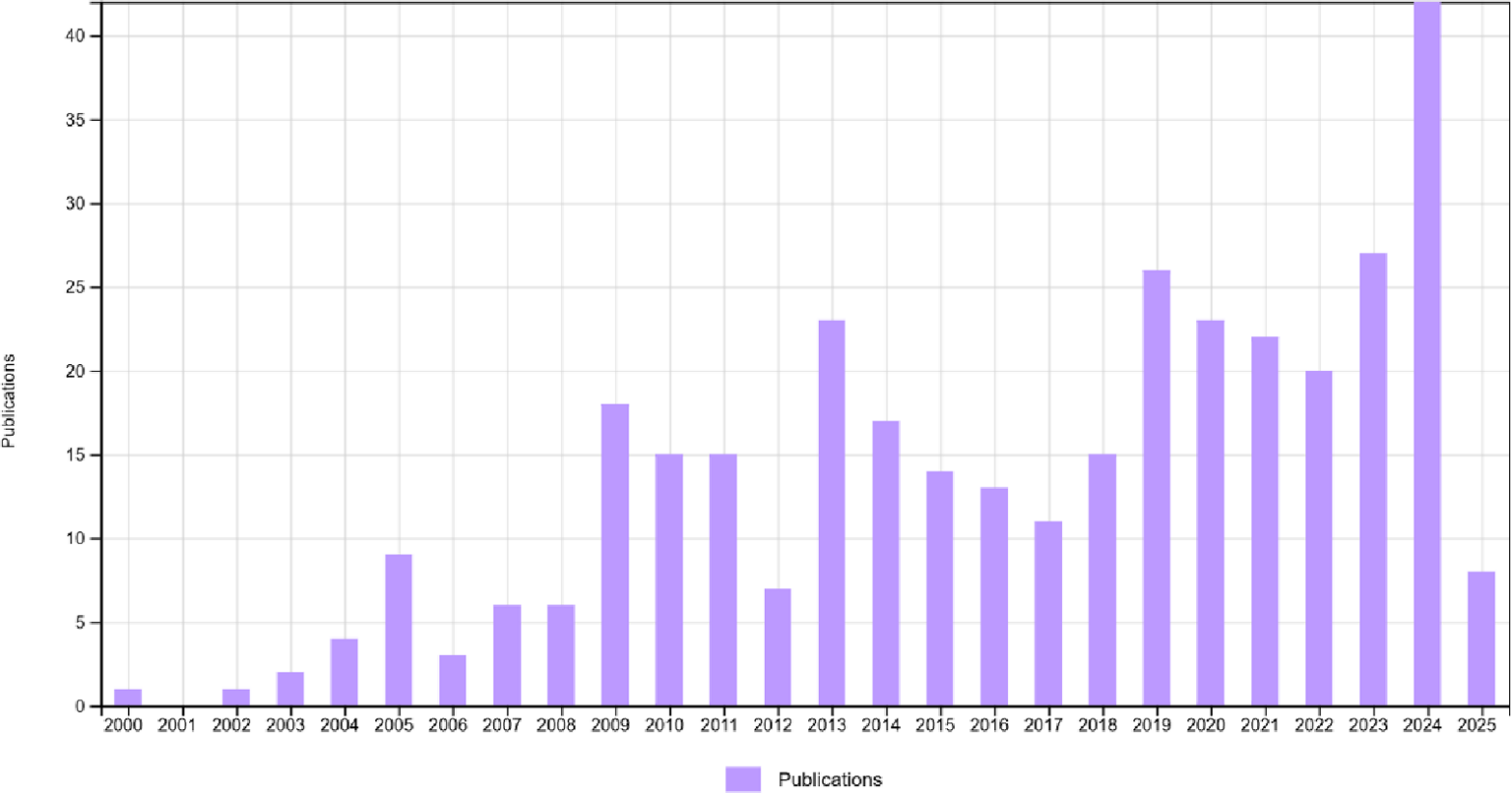
Publications summary. Bar chart showing the yearly distribution of publications on workplace coaching from 2000 to 2025, highlighting trends and growth patterns in the academic literature.

This research depicts the annual publication and citation summary of workplace coaching from 2000 to 2025 (June) in Figure 2. At the outset, the number of publications began with one in 2000, and in 2001, there were no publications. The graph increased from 0 to 18 in 2009 and further rose to a maximum of 42 publications in 2024. The graph indicates that in 2024, researchers identified workplace coaching as a key area for growth and development. This sudden shift in the research area could be attributed to employees’ high involvement and engagement with technology, organizational expectations, leadership identity, and well-being, or the need to explore the importance and uniqueness of coaching in the workplace at various levels ( Alves & Figueiredo, 2024; Cannata & Danels, 2024; Harris, 2024).

Furthermore, this study provides a summary of prominent publication sources in the domain of workplace coaching. Journals play a crucial role in providing a platform for scholarly articles and serve as an academic reserve for future research, offering foundational ideas. Journal metrics, such as the impact factor, citation score, H index, and G index, among others, serve as vital indicators to define the quality of a journal. A total of 343 research articles were considered for this study, of which 129 publication titles and 38 publication houses were included in the final search. The most prominent journal publishing houses include Emerald, Wiley, HBR, Sage Inc., and Frontiers Media, which are home to the top 10 publishing titles, as shown in Table 5.

**Table 5.** Summary of prominent sources.

Publication titles	Record count	Publisher	Total citations	Impact factor	H Index
Journal of Management Development	18	Emerald Group Publishing Ltd	361	3.4	8
Frontiers in Psychology	12	Frontiers Media	95	2.6	5
Human Resource Development Quarterly	9	Wiley	194	4	6
International Journal of Mentoring and Coaching in Education	9	Emerald Group Publishing Ltd	60	1.9	4
Harvard Business Review	8	Harvard Business School Publishing Corporation	492	9.1	6
Journal of Applied Behavioural Science	7	Sage Publications Inc	97	3.9	5
Advances in Developing Human Resources	6	Sage Publications Inc	101	3.3	5
Journal of Educational Administration	6	Emerald Group Publishing Ltd	129	2.7	5
Journal of Occupational and Organisational Psychology	5	Wiley	264	6.2	1
Leadership Organisation Development Journal	5	Emerald Group Publishing Ltd	37	5	4

Note - This table lists major publication sources relevant to coaching research, including journal titles, publication counts, publishers, citation totals, impact factors, and H-indices. It provides an overview of key journals contributing to the academic impact and dissemination in the field.

**Figure 3.**
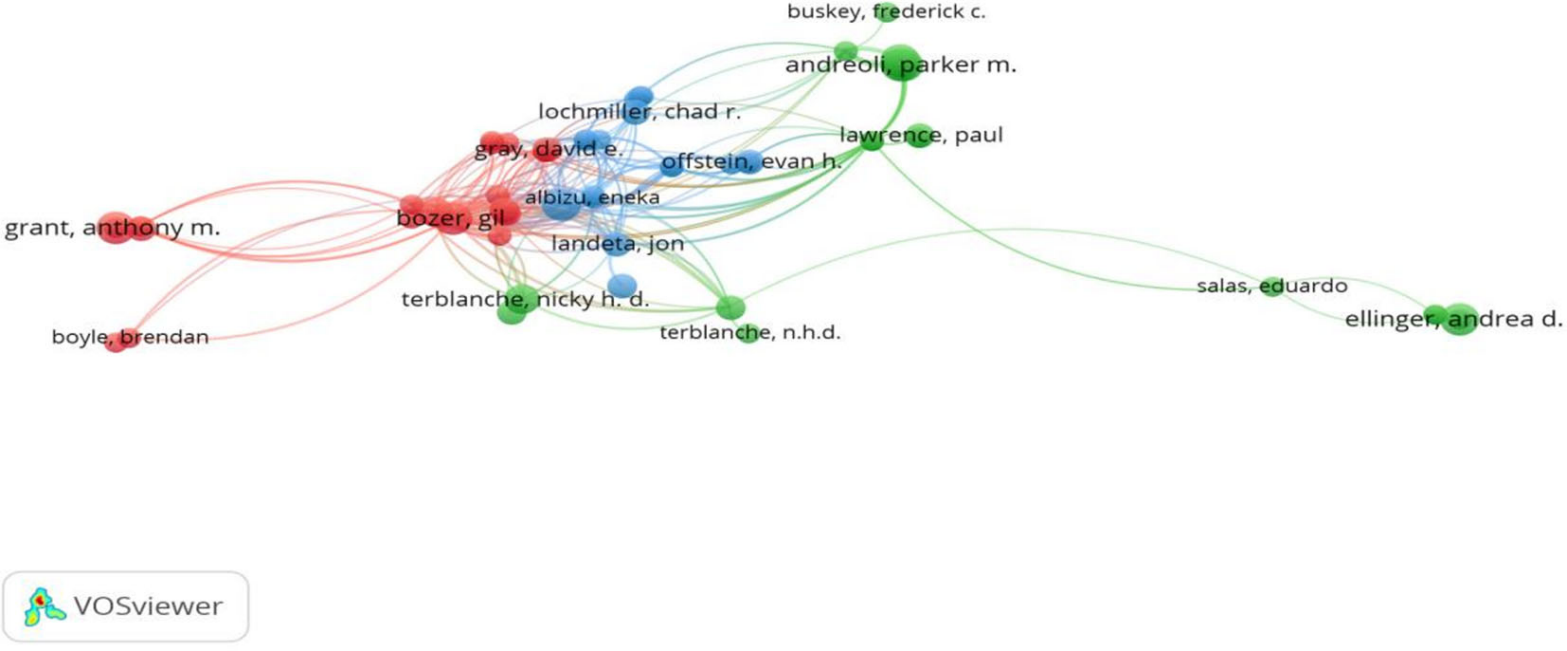
Most prominent authors and citation networks. Network visualization displaying key authors and citation relationships within the field, mapping influential research collaborations and scholarly clusters in workplace coaching.

Using the number of published research articles and the total number of citations as a base, this study identified the most prominent authors in the workplace coaching domain. Among the 527 authors in the study area, Terblanche N published 10 articles with 271 citations related to executive coaching, workplace coaching, diversity, and the potential impact of artificial intelligence on humanity ( Kruger & Terblanche, 2024; N. Terblanche, 2020; N. H. D. Terblanche, 2022). At the second level, Bozer G has eight publications, with the second-highest citations of 406, primarily focused on workplace coaching, incorporating organizational psychology into theory building and systematic literature reviews ( Bozer & Jones, 2018, 2021). In the next line, Klar Hans W, Gray David E, Jones Rebecca J, with 308, 169, and 446 citations, respectively, made significant contributions in the areas of leadership coaching, executive coaching, employee coaching, and coach development using personality models, organizational psychology, and transformative leadership approaches ( Bozer & Jones, 2018, 2021; Carden et al., 2022; R. J. Jones et al., 2016; Klar et al., 2020). Other noteworthy authors include Andrea D. Ellinger and Kristin Shawn Huggins, who discussed leadership coaching and human resource development ( A. D. Ellinger & Kim, 2014; A. E. Ellinger & Ellinger, 2014), as shown in Table 6 and Figure 3.

**Table 6.** Summary of prominent author profiles.

Researcher profiles	Count	Affiliation	Total citations
Terblanche, Nicky	10	Stellenbosch Business Sch Stellenbosch University	271
Bozer, Gil	8	Sapir Academic Cell, Monash University	406
Klar, Hans W.	6	Clemson University, Sydney Inst Language & Commerce, University of Technology Sydney	308
Gray, David E.	6	University of Greenwich	169
Jones, Rebecca J.	6	Henley Business School, University of Reading	446
Andreoli, Parker M.	5	Educational & Organisational Leadership Development College of Education, Clemson University	39
Ellinger, Andrea D.	5	The University of Texas at Tyler	157
Huggins, Kristin Shawn	5	Washington State University	39
Cilliers, Frans V	5	University of South Africa	37
Offstein, Evan	4	Frostburg State University	15

Note - This table highlights leading researchers in the coaching field, indicating their publication counts, institutional affiliations, and total citation figures. It showcases key contributors and their impact within the academic research community.

**Figure 4.**
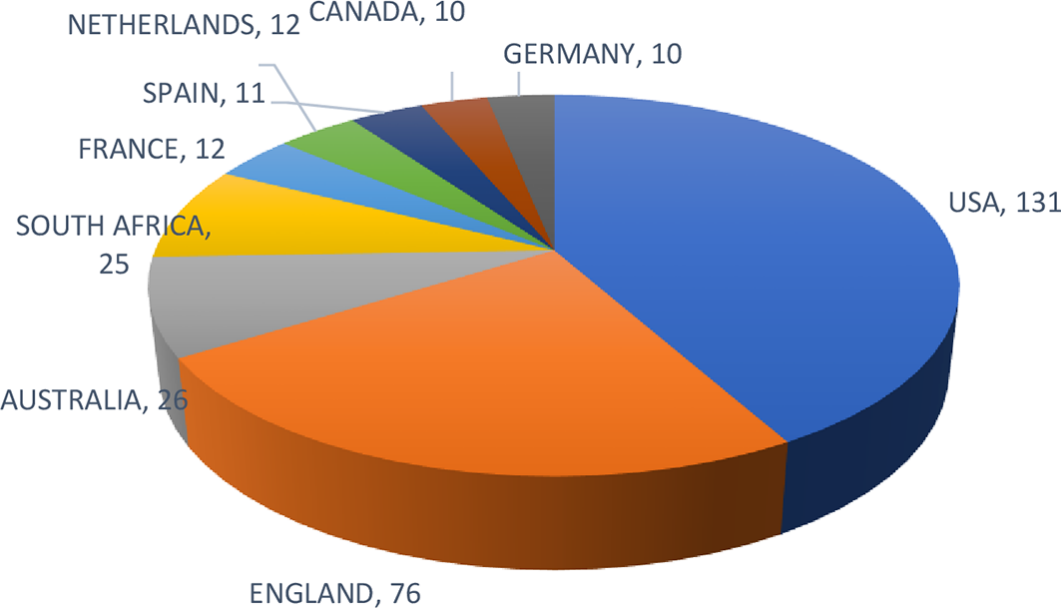
Most prominent countries. Pie chart presenting the distribution of workplace coaching research by country, indicating the relative contribution of major countries to the global literature base.

The top 10 prominent countries in this study are shown in Figure 4. The United States of America is the most prominent country, with 131 documents, followed by England (76) and Australia (26). Authors from these countries have initiated the concepts of leadership, executive, and employee coaching as foundational and review papers. ( Day, 2000; Feldman & Lankau, 2005; Heslin et al., 2006).

Table 7 summarizes the most prominent and foundational research works in the area of workplace coaching, including the total citation count and the average number of citations per year. The most prominent and foundational article in workplace coaching, published in 2000 by Day as a review paper, highlighted the need for leadership development through coaching as an intervention ( Day, 2000). In later stages, the idea of employee coaching became prominent because of the concept of talent management, written by Cappelli in 2008 and published by the Harvard Business Review ( Cappelli, 2008). Introduction to the idea of executive coaching was given in 2009 by Grant et al., in association with goal attainment, resilience, and workplace well-being, became one of the top three publications in the list, and ( Grant et al., 2009). Later, in continuation with the three research works, Jones and team conducted a meta-analysis to understand the effectiveness of workplace coaching ( Jones et al., 2016). The preference for person theories and their support for employee coaching has been studied further by Heslin et al., (2006).

**Table 7.** Summary of foundation articles in workplace coaching.

Title with author details	Total citations	Avg citation per year
Leadership development: A review in context ( Day, 2000)	1077	41.42
Talent management for the twenty-first century ( Cappelli, 2008)	271	15.06
Executive coaching enhances goal attainment, resilience, and workplace well-being: a randomized controlled study ( Grant et al., 2009)	265	15.59
The effectiveness of workplace coaching: A meta-analysis of learning and performance outcomes from coaching ( Jones et al., 2016)	263	26.3
Keen to help? Managers’ implicit person theories and their subsequent employee coaching ( Heslin et al., 2006)	249	12.45

Note - This table lists the most highly cited publications in the coaching and leadership domain, providing full citation details, total number of citations, and average citations per year, thereby highlighting foundational works with significant scholarly impact.

### 4.1 Keywords and cluster analysis

This section examines the most relevant author keywords based on occurrence scores and total link strengths. Executive coaching is considered the most repeated and relevant among all workplace coaching research, occurring 83 times, with 72 total link strengths. This was followed by coaching, leadership development, workplace coaching, leadership coaching, and others ( Table 8).

**Table 8.** Most relevant author keywords.

Rank	Keyword	Occurrence	Total link strength
1	Executive coaching	83	72
2	Coaching	76	87
3	Leadership development	28	42
4	Leadership	22	26
5	Workplace coaching	17	17
6	Leadership coaching	15	13
7	Management development	15	24
8	Coaching effectiveness	13	20
9	Managerial coaching	9	15
10	Employee coaching	8	5

Note - This table ranks the top research keywords in the coaching and leadership domain by frequency of occurrence and total link strength, mapping thematic prominence and connectivity within scholarly publications.

**Figure 5.**
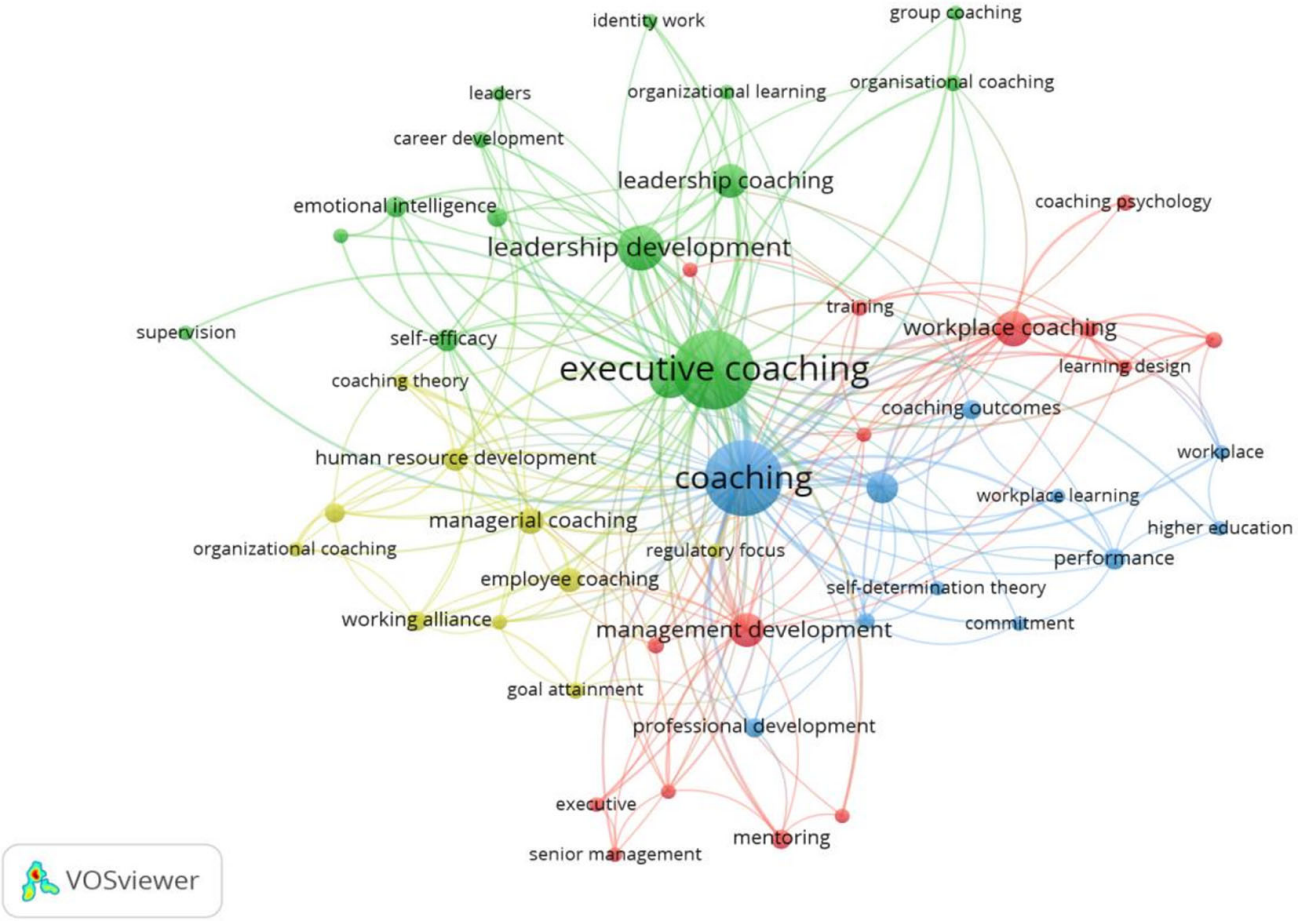
Network visualization. Co-occurrence network visualization of prevalent keywords in workplace coaching research, revealing thematic clusters and interconnections among core concepts.

**Figure 6.**
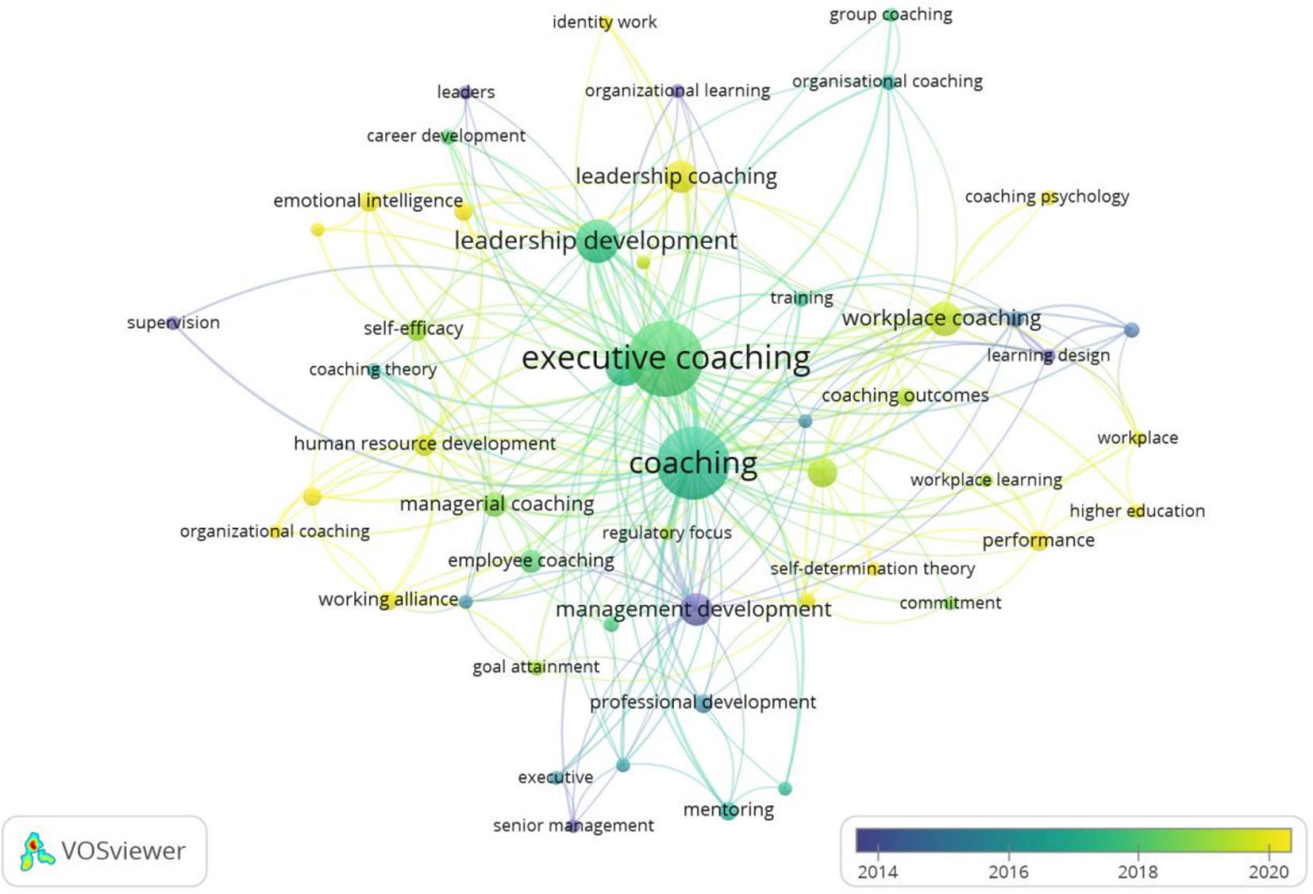
Overlay visualization. Temporal overlay visualization of keyword co-occurrence networks, illustrating shifts in research focus and the emergence of new themes in workplace coaching over time.

This study leveraged the author’s logical understanding, theoretical integration, and descriptive acumen to evaluate the clusters in workplace coaching research. Four clusters were explored and themed, based on the foundational research ideas identified in. Figures 5 and 6 show the co-occurrence network and overlay visualizations, respectively.

The first cluster is themed based on the keyword integration towards “ *Coaching for Workplace Learning and Development cluster*,” which is identified in Red with 15 items. In this cluster, researchers initially studied keywords such as learning design, management development, and organizational development concepts. Later, the literature evolved to include workplace coaching, coaching psychology, mentoring, training, and related concepts. According to Cannon-Bowers et al. (2023), workplace coaching has emerged as the most popular research idea over the last two decades and is considered one of the fastest-growing performance enhancement tools within organizations. During 2014, the term “workplace coaching” was not very prominent, and most studies were inclined towards training, learning designs, and development for senior management and executives ( Crawford & Earley, 2011; A. E. Ellinger & Ellinger, 2014; O’Connor & Cavanagh, 2013). Workplace coaching was considered a rebuilding strategy in organizations ( Offstein et al., 2013) and advanced through a work alliance relationship between the coach and the coachee, utilizing psychological theories as shown in the overlay visualization ( Kruger & Terblanche, 2024). In summary, the literature and sub-themes reveal that workplace coaching offers psychological benefits, including personal growth and professional development, increased motivation levels, and enhanced loyalty towards organizations. Further coaching strategies include leader development programs and tailored coaching practices for individuals and groups, ultimately leading to performance enhancement and sustainable well-being. Overall, coaching contributes to organizational performance and employee retention by directly influencing job performance and commitment towards work and the organization.

The most transformative and centralized cluster (green) is the second one, identified as “Multifaceted advantage of Executive and Leadership Coaching,” comprising 15 items. These items encompass both leadership and executive coaching concepts, including identity, leadership, career development, self-efficacy, emotional intelligence, group, and organizational coaching. The initial set of studies primarily focused on the effectiveness of executive coaching in leadership development ( Kombarakaran et al., 2008) where it is considered a valuable tool in enhancing managerial flexibility and leadership behaviors ( R. A. Jones et al., 2006). Transformative learning concepts have gained attention in understanding how executive coaching Studies fosters critical reflection and rational dialogue ( Mbokota et al., 2022). Strength-based coaching significantly increases transformational leadership behaviors among executives ( MacKie, 2014). Executive coaching started to be examined as an intervention to drive gender diversity in leadership roles, mainly focused on women in leadership positions ( Maher & Hastings, 2023). The role of identity work in leadership coaching is identified as a critical factor in effective coaching outcomes ( Szekely et al., 2025). These developments in executive and leadership coaching reflect a broader understanding of the multifaceted impact on leadership development and organisational change.

The third (blue) cluster is *titled “Dimensions of Workplace Coaching” and comprises 11 items that are inclined towards various research ideas on coaching effectiveness, commitment, and outcomes.* Workplace coaching has shown a significant improvement in individual-level outcomes, including performance, skills, wellbeing, coping mechanisms, and goal-directed attitudes ( Theeboom et al., 2017), and positively affects organizational outcomes, including skill-based, affective, and individual-level results ( R. J. Jones et al., 2016). Workplace coaching enhances team commitment, which in turn improves team performance ( Weer et al., 2016), Tailored coaching strategies are believed to be the most effective and successful interventions for employees and managers ( Longenecker, 2010). Coaching outcomes are measured at the individual, group, and organizational levels based on the goals set by the coach and leaders ( Habig & Hoole, 2016). External coaches and blended coaching formats are strongly associated with well-being outcomes at leadership levels ( R. J. Jones et al., 2018). In summary, the workplace coaching dimensions are identified as effectiveness, commitment and multi-level outcome measurement in achieving successful coaching interventions shown in this cluster.

The last and fourth cluster (Yellow) is “ *Employee Coaching for Human Resource Management and development*” with 10 items including employee coaching, managerial coaching, human resources management, development, working alliance, coaching theories and more. Coaching in the workplace has a significant impact on employee performance, and studies have shown that coaching can show a variance in performance between 2.9% and 6.2% when controlling for tenure and experience ( Pousa & Mathieu, 2014). Managerial coaching affects work engagement, improves leader-member exchange quality and job satisfaction, and reduces turnover intentions ( Ali et al., 2018). Employee coaching increases individual propensity for self-development and self-improvement, which in turn boosts their efficiency ( Askhatova et al., 2015). Mentoring and employee coaching provide greater visibility and access to information and resources, leading to higher performance, satisfaction, and productivity ( Peláez Zuberbühler et al., 2023) consistent across different career stages ( Pousa et al., 2017). Overall, employee coaching is a valuable tool for employee performance, development, and satisfaction across various sectors and career stages.

## 5. Discussion

This study employed a systematic literature review, utilizing bibliometric techniques, to examine trends and advancements in workplace coaching. We consider both the SCOPUS and WoS databases, citation reports, and various bibliometric methods to provide insights into the current state of existing works and the progression of workplace coaching research over time. While the most recent study by Passmore et al., (2025) has consolidated both empirical and conceptual findings of peer-reviewed research articles on the use of AI in coaching; however, the primary focus of the study is on a qualitative approach and lacks quantification of research trends. Another review paper Kapoutzis et al., (2024) on the detangling of definitions and conditions under which coaching culture can be developed using the SLR technique; however, the researchers agreed that the foundational elements, interventions, measures, and outcomes were still ambiguous and required a more quantitative understanding.

Another study Wang et al., (2022) conducted a meta-analysis with robust variance estimates and confirmed that psychologically informed coaching approaches had a positive effect on goal attainment and self-efficacy. Lai & Palmer (2019) conducted a similar study on psychologically informed coaching approaches using qualitative and quantitative methods by consulting ten experts in the field to establish empirical support. While their studies effectively conceptualized the idea of effective coaching approaches to facilitate desirable organizational outcomes using meta-analysis, they did not map the scholarly networks and citation patterns that shape the field’s intellectual landscapes. Gawlik et al., (2023) studied coaching interventions aimed at increasing physical activity and health behavior in the workplace, which have shown positive outcomes using SLR and PRISMA guidelines; however, the variety of coaching parameters and their effectiveness remain inconclusive without quantitative analysis, and suggest a need for more standardized approaches.

Exclusive bibliometric studies were also conducted by Tewari et al. (2025) on flexible work arrangements in learning organizations and by Mahdi et al. (2024) on navigating the landscape of academic coaching; however, both studies focused on the bibliometric landscape of learning organizations and academic coaching, rather than workplace coaching. Therefore, the present study complements these studies by applying bibliometric techniques and methods to systematically examine the field’s development, influential authors and research works, and their collaborations and future trends in detail. In addition, the current study extends its scope by examining the uniqueness and differences in workplace coaching terminologies, including leadership coaching, executive coaching, and employee coaching. In addition, the study also included the evolution of these key themes and sub-themes theoretically using a systematic literature review and empirically using science mapping techniques, as shown in Table 9.

**Table 9.** Summary of key themes, concepts, relationships, and findings of workplace coaching.

Cluster	Key themes	Key concepts	Relationships	Findings	References
Cluster 1 - Red with 15 author keywords	Coaching for Workplace Learning and Development	Management development, workplace coaching, coaching psychology, learning design, training, organisational development, mentoring, resilience, senior management	Workplace coaching and learning theories --> enhances effectiveness at the workplace; Managerial and leadership development --> team development and organisational performance	Workplace coaching improves performance, well-being and adaptability among employees; Key psychological mechanisms like self-regulation and growth mindset significantly contribute to coaching effectiveness; Tailored coaching strategies to individual needs enhance its impact.	( Bakhshandeh et al., 2023; Berg & Karlsen, 2012; Cannon-Bowers et al., 2023; R. J. Jones et al., 2016; Lyons & Bandura, 2021; Rajasinghe & Allen, 2020)
Cluster 2 - Green with 15 author keywords	Multifaceted advantage of Executive and Leadership Coaching	Executive coaching, leadership coaching, leadership development, organisational coaching, group coaching, self-efficacy	Executive coaching --> self-efficacy; Leadership development --> organisational impact; group coaching --> intra-organisational cooperation and economic results; Leadership competencies --> career satisfaction	Positive impact of multiple coaching sessions on self-efficacy; Improvement in people management, goal setting, engagement, and communication; Enhanced creativity, innovation, flexibility, and resilience; Benefits include problem-solving, self-awareness, self-confidence, and cooperation; Improved job performance, personal vision, engagement, and career satisfaction.	( Alves & Figueiredo, 2024; Alvey & Barclay, 2007; Baron & Morin, 2010; Brooks et al., 2023; Grant, 2014; Kombarakaran et al., 2008; Van Oosten et al., 2019)
Cluster 3 - Blue with 11 author keywords	Dimensions of Workplace Coaching	Coaching effectiveness, coaching outcomes, commitment, motivation, professional development, self-determination	Coaching effectiveness --> coaching outcomes (performance, wellbeing, coping, work attitudes, goal-directed self-regulation); affective commitment --> work alliance; Individualised strategies --> specific development needs; Leadership development --> communication and relationship building	Effectiveness is influenced by coach professionalism, salary satisfaction and training; coaching outcomes improve performance, wellbeing, and self-regulation; Coaching commitment is enhanced through strong working alliances and affective commitment; Self-determination is supported by autonomy, competence and relatedness.	( Bozer et al., 2013; Bozer & Jones, 2018; Chong et al., 2016; Fingas et al., 2025; R. J. Jones et al., 2016, 2018; Theeboom et al., 2017)
Cluster 4 - Yellow with 10 author keywords	Employee Coaching for Human Resource Management and Development	Coaching relationship, coaching theory, goal attainment, human resource development, human resource management, regulatory focus, and working alliances	Coaching relationships --> employee perceptions and behaviours; coaching theories --> directive and non-directive approaches and implicit person theories; Goal attainment --> individual strategies and performance improvement; HRD--> creating coaching culture --> integration in HRM	Coaching enhances skills, knowledge, and attitudes, improving work quality and customer satisfaction. Integration of coaching in HRM supports talent strategies and creates a coaching culture. Coaching leadership enhances engagement through organisational self-esteem and learning goal orientation. Strong working alliances are essential for effective coaching outcomes.	( Bakhshandeh et al., 2023; Gladis, 2007; Liu et al., 2024; Pousa et al., 2017; Pousa & Mathieu, 2014)

Note - This table presents the major thematic clusters identified in coaching literature, summarizing key themes, central concepts, core relationships, empirical findings, and primary references. Each cluster showcases distinct research foci and the supporting evidence base underlying coaching theory and practice.

A rigorous bibliometric analysis was employed in this study to examine publications and citation trends from 2000 to 2025, utilizing the citation analysis technique. In addition, the study examined the most prominent publication titles, foundational articles, and influential authors in the field of workplace coaching. Furthermore, the most influential countries with established affiliations were examined to understand the significance of workplace coaching globally. This study adopted a prominent and holistic approach by integrating co-authors and co-occurrence analyses to uncover collaborations and thematic clusters in workplace coaching. To offer a comprehensive and forward-looking perspective on workplace coaching research, this study employed key metrics, including citation patterns and co-authorship networks, to identify research trends. Keyword analysis was employed to identify influential research themes and network maps that revealed collaboration patterns, ensuring the study’s findings aligned with its objectives.

To conduct a bibliometric analysis ethically, maintaining data transparency is critical in this research type. Therefore, this study adhered to all the standards and guidelines set by the PRISMA framework, which includes the identification, acquisition, organization, purification, evaluation, and reporting of data, as shown in Figure 1. Thus, this study not only extends the current understanding of workplace coaching research but also sets the foundation for future research. The practical and theoretical implications of this study serve as a key resource for scholars, practitioners, and policymakers seeking to enhance employee, leadership, and executive coaching experiences across various sectors and diverse settings.

## 6. Practical and theoretical implications

The current dynamic business environment, characterized by volatility, uncertainty, complexity, and ambiguity (VUCA), necessitates the implementation of workplace coaching to help organizations and employees adapt and thrive. Coaching is a promising method for enhancing employee resilience, which is essential for navigating adversity in a demanding workplace ( Sipondo & Terblanche, 2024). Customized coaching strategies help employees manage their emotions, find meaning in challenging situations, and develop skills to overcome difficulties in uncertain situations ( Jouali et al., 2024). Supportive leadership coaching and coworker support are positively associated with employee resilience, especially under high work pressure and in target jobs, highlighting a serious need for workplace coaching to support employee resilience and well-being. The implications of the bibliometric and systematic literature review of workplace coaching are twofold and are relevant for both the academic community and organizational practitioners.

For the academic community, this study identified the foundational understanding of workplace coaching, its uniqueness and differences, theoretical contributions, seminal works, influential authors, research documents, and keywords. This provides a comprehensive framework for advancing academic discourse on coaching in workplace research, enabling the most targeted and impactful future investigation. By contrast, for practitioners, this study offers human resource management and organizational development support, providing actionable insights into the global landscape of workplace coaching. Regional and geographical analysis, key institutional affiliations, and emerging research trends in workplace coaching enable organizations to develop effective strategies for enhancing workplace well-being and resilience. This knowledge can guide practitioners in developing tailored coaching interventions to address specific workplace challenges and issues at various levels. Similarly, fostering a supportive and positive workplace for individuals, groups, and organizational representatives.

## 7. Conclusion, future direction, and limitations

This systematic literature review article attempts to trace the evolution and progression of workplace coaching research while also suggesting potential future directions. The findings of this research revealed an increasing publication and citation trend after 2020, particularly in 2024, with 42 publications and 925 citations, indicating an anticipated rise in research contributions in the years to come. The study further identified key publication titles, “Journal of Management Development” and “Frontiers in Psychology,” as the most influential journals in workplace coaching research. Furthermore, Terblanche, Nicky, and Bozer, Gil, as influential authors, and the USA and England, as influential countries, are notable. The articles “Leadership development: A review in context” ( Day, 2000) and “Talent management for the twenty-first century” ( Cappelli, 2008) are among the most influential, with 1077 and 271 total citations, respectively. Moreover, executive coaching, coaching, leadership development, employee coaching, and leadership coaching were identified as the most common keywords in author keyword searches. The keyword co-occurrence analysis identified four clusters: Coaching for Workplace Learning and Development (red), multifaceted advantages of Executive and Leadership Coaching (green), Dimensions of Workplace Coaching (blue), and Employee Coaching for Human Resource Management and Development (yellow), setting the stage for future direction and practical implications aimed at enhancing and improving workplace coaching interventions, refer to Table 10.

**Table 10.** Summary of past, present and future of workplace coaching.

What is extensively studied in workplace coaching research?	What areas has this study explored as gaps?	What are the suggested future areas in workplace coaching from this study?
Coaching effectiveness – Coaching positively impacts skill-based, affective, and individual level outcomes ( Bozer & Jones, 2018; Cannon-Bowers et al., 2023; de Haan & Nilsson, 2023; R. J. Jones et al., 2016)	Learning process and goal attainment (Cluster 1) - interventions like clarification of meaning and mastery/coping have shown significant effects on goal attainment, emphasising the importance of stimulating learning processes in coaching ( Antoni & Tatar, 2025)	**Diversity and inclusion in workplace coaching** – Explore the effectiveness of coaching for underrepresented groups and its role in fostering inclusive leadership practices (Cluster 3).
Determinants of Workplace Coaching - self-efficacy, coaching motivation, goal orientation, trust, interpersonal attraction, feedback intervention, and supervisory support ( Bozer & Jones, 2018); The involvement of managers, particularly discretionary effort, is perceived to enhance coaching outcomes ( Webster, 2018)	Effectiveness and success factors of executive coaching (Cluster 2) - Improve personal and organizational outcomes, such as self-awareness, career satisfaction, organizational commitment, and task performance ( Bozer et al., 2015; Calasso et al., 2024); specific behaviours and phases of coaching, informed by models like GROW, are crucial for success ( Bozer et al., 2015)	**Integration of technology in coaching** – Evaluate the effectiveness of virtual coaching sessions compared to traditional face-to-face coaching, particularly in hybrid work environments. **Coaching for change management** – Investigate the role of coaching in supporting organizational change initiatives (Cluster 2).
Types of coaching - Internal coaches tend to be more effective than external coaches, especially in complex job roles ( R. J. Jones et al., 2016, 2018); Blended coaching formats (face-to-face combined with e-coaching) are associated with better well-being outcomes ( R. J. Jones et al., 2018)	Leadership Development (Cluster 2) - Executive coaching significantly enhances leadership skills, including people management, goal setting, engagement, and communication ( Kombarakaran et al., 2008); impact on leadership behaviour ( Anthony, 2017; Lochmiller, 2014); c-suite transitions ( McGill et al., 2019) and developmental value ( Brown et al., 2021)	**Coaching for resilience and well-being ** – Assess the role of coaching in preventing burnout and promoting mental health in the workplace **Coaching in Remote and Hybrid Work Settings** – Explore how coaching can facilitate team cohesion, communication, and performance in distributed teams (Cluster 4)
Barriers to effective coaching - Common barriers include difficulties with the coach, coaching relationships, and overall coaching experience and lack of organisational support is not necessarily predictive of limited coaching effectiveness ( Webster, 2018)	Employee Coaching (Cluster 4) - improves frontline employee performance across all career stages; participative management style, enhancing team-member engagement and reducing turnover intentions ( Pousa & Mathieu, 2014); Managerial Coaching Assessment System (MCAS) and Rational Managerial Coaching Program (rMCP) enhance managerial coaching skills ( DiGirolamo & Tkach, 2019)	**Coaching models and framework** – Develop and validate new coaching models that integrate psychological theories and evidence-based practices **Coaching supervision and ethics** – Examine the role of supervision in coaching practices and the ethical considerations involved (Cluster 1)
Coaching trends – third-generation coaching trends and evidence-based coaching trends were opted to differentiate from pseudoscientific practices ( Grant, 2014; Grant & Cavanagh, 2007)	Dimensions of coaching (Cluster 3) - success criteria for coaching, such as trust, acceptance, and commitment to the coaching process ( de Haan, 2019); stimulates learning processes; mediates the impact on goal attainment ( Antoni & Tatar, 2025)	**Coaching outcome beyond performance** – Investigate the broader outcomes of coaching, including personal development, emotional intelligence, and interpersonal relationships (All cluster keywords)

Note - This table synthesizes key research trends, identified gaps, and proposed future directions in workplace coaching. It highlights well-established findings, addresses unexplored areas, and suggests topics such as diversity and inclusion, technology integration, resilience, and new coaching models for further investigation.

In alignment with the bibliometric findings presented in this study, the conclusions are grounded in measurable evidence. The observed publication growth trend—rising from 1 article in 2000 to a peak of 42 publications in 2024—demonstrates a statistically significant upward trajectory in research interest in workplace coaching. Dominant keywords identified through co-occurrence analysis, namely executive coaching (83 occurrences, 72 total link strength), leadership development, and workplace coaching, confirm that leadership and executive coaching remain the primary thematic concerns of the field. The four thematic clusters derived from keyword co-occurrence (red: Coaching for Workplace Learning and Development; green: Multifaceted Advantages of Executive and Leadership Coaching; blue: Dimensions of Workplace Coaching; yellow: Employee Coaching for HRM and Development) provide an evidence-based map of the field’s intellectual structure. Highly cited works by ( Cappelli, 2008); ( Day, 2000) and; ( Grant et al., 2009) —accumulating 1077, 271, and significant citation counts respectively—anchor the conclusions regarding foundational contributions to workplace coaching research. All interpretive claims in this study are thus bounded by and traceable to the bibliometric outputs described above.

### 7.1 Key evidence-based findings

The following findings are directly supported by the bibliometric data collected and analyzed in this study. Publication and citation trends confirm a steady and accelerating growth in workplace coaching research from 2000 to 2025, with the most productive year being 2024 (42 publications, 925 citations). Among 527 contributing authors, (Terblanche, 2022) Terblanche N (10 articles, 271 citations) and Bozer G (8 articles, 406 citations) are the most prolific and cited contributors (Bozer et al., 2013, 2015; Bozer & Jones, 2018a, 2018b, 2021). The United States (131 documents), England (76), and Australia (26) are the leading countries in workplace coaching research. Co-occurrence analysis confirms that executive coaching, coaching, leadership development, workplace coaching, and leadership coaching are the five most frequent and interconnected author keywords. These findings collectively establish that workplace coaching research is dominated by Anglo-American scholarship, with a strong orientation toward leadership and executive contexts.

### 7.2 Future research directions

Building on the bibliometric evidence above, and clearly distinguished from the data-driven findings, the following directions are proposed for future inquiry. Under-researched themes identified in the overlay visualization—including artificial intelligence in coaching, ethical considerations, digital transformation, and sustainability in leadership—represent emergent topics with limited citation counts that merit dedicated investigation. Regions underrepresented in the co-authorship network, such as South Asia, Sub-Saharan Africa, and Latin America, offer opportunities for cross-cultural and comparative studies. Future studies may also employ meta-analyses incorporating grey literature to provide a more comprehensive evaluation of coaching effectiveness across sectors and career stages. Longitudinal mixed-method designs could complement the current bibliometric baseline by capturing the dynamic evolution of coaching practices over time.

## Corresponding author details

Dr Itam Urmila Jagadeeswari

Affiliation – Dept of Commerce, Manipal Academy of Higher Education, Bangalore

Contact Information – urmilaitam@gmail.com; urmila.itam@manipal.edu

## Declaration on the use of AI statement

The authors affirm that no generative Artificial Intelligence (AI) tools or technologies were used in the conceptualization, writing, analysis, or interpretation of data for this research study. All aspects of the research, including literature review, methodology design, data collection, data analysis, and manuscript preparation, were conducted solely by the authors. This declaration ensures the authenticity, originality, and academic integrity of the research presented.

## Data Availability

This study is a systematic literature review of workplace coaching. High-quality publications were considered for review. Qualitative analysis was conducted using the PRISMA framework and VOSviewer software to generate the items and relevant themes. This work contains the following underlying data: Itam, Urmila Jagadeeswari; Shukla, Nidhi; Toni, Mercy; Keerti Mishra, Ar. (2025). **A Systematic Literature Review and Bibliometric Analysis of Workplace Coaching**. figshare. Journal contribution. https://doi.org/10.6084/m9.figshare.30381517 ( Itam et al., (2025)). Data are available under the terms of the Creative Commons Attribution 4.0 International license (CC-BY 4.0).
