# Supplementary material for: A Systematic Literature Review and Bibliometric Analysis of Workplace Coaching
Source: F1000Res. 2026 Apr 29;14:1290. Originally published 2025 Nov 21. [Version 2] doi: 10.12688/f1000research.171808.2 (PMC13202203; doi:10.12688/f1000research.171808.2)
Supplement: Peer review history

# Reviewer report: Reviewer response for version 2

**DOI:** 10.5256/f1000research.198611.r480892

**Published:** 2026-06-05

**Authors:** Gnanendra M

**Affiliations:** 1 Faculty of Management Studies, CMS Business School, JAIN (Deemed to Be University), Bengaluru, Karnataka, India

The manuscript presents a thorough, well-organized SLR and bibliometric analysis of workplace coaching. The topic is relevant and timely because coaching practices are increasingly a part of organizational development and employee performance.

 The study is methodologically sound, as a systematic review approach was followed and bibliometric techniques were used to identify influential authors, sources, themes, and research trends. The analysis is well organized, and the results provide a good understanding of the development and status of research on workplace coaching. The discussion summarizes the literature and outlines directions for further research.

 The manuscript is a valuable contribution to the field because it brings together diverse knowledge and offers a practical reference for researchers and practitioners interested in workplace coaching. The scientific quality and the validity of the study may be improved by a few minor editorial changes, but these do not alter the message.

 The article is well written overall, the conclusions are well supported by the evidence presented, and it is appropriate for indexing.

 The current draft is a scientifically valid article. The methodology is suitable, the results are well presented, and the conclusions are supported by analysis. Any recommended changes are minor and not critical.

Are the rationale for, and objectives of, the Systematic Review clearly stated?

Yes

Is the statistical analysis and its interpretation appropriate?

Partly

If this is a Living Systematic Review, is the ‘living’ method appropriate and is the search schedule clearly defined and justified? (‘Living Systematic Review’ or a variation of this term should be included in the title.)

Not applicable

Are sufficient details of the methods and analysis provided to allow replication by others?

Yes

Are the conclusions drawn adequately supported by the results presented in the review?

Yes

Reviewer Expertise:

Management, Bibliometric Analysis, Financial Econometrics, Financial Inclusion

I confirm that I have read this submission and believe that I have an appropriate level of expertise to confirm that it is of an acceptable scientific standard.

# Reviewer report: Reviewer response for version 2

**DOI:** 10.5256/f1000research.198611.r480897

**Published:** 2026-05-25

**Authors:** Thangaraja Arumugam

**Affiliations:** 1 Vellore Institute of Technology - Chennai Campus, Chennai, Tamil Nadu, India

The manuscript titled *“A Systematic Literature Review and Bibliometric Analysis of Workplace Coaching”* is a well-structured and meaningful contribution to the workplace coaching literature. The study addresses a highly relevant and contemporary topic with clear research objectives and a strong theoretical foundation.

 The authors have adopted a systematic and rigorous methodology using PRISMA guidelines, Scopus and Web of Science databases, and bibliometric tools such as VOSviewer. The search strategy, inclusion criteria, and analysis procedures are clearly explained, making the study transparent and replicable.

 The literature review is comprehensive and effectively differentiates leadership coaching, executive coaching, and employee coaching. The bibliometric analysis, keyword co-occurrence mapping, thematic clustering, and citation analysis are appropriately conducted and well interpreted. The identification of four major thematic clusters provides valuable insights into the evolution and future direction of workplace coaching research.

 The discussion and conclusion are well supported by the results presented in the study. The practical implications for organizations, HR professionals, and leadership development are also clearly highlighted. The manuscript further provides useful future research directions in areas such as AI-enabled coaching, digital transformation, employee well-being, and inclusive leadership.

 Overall, the manuscript demonstrates good academic quality, methodological rigor, and practical relevance. The study makes a significant contribution to the field and is suitable for publication.

 I recommend the manuscript for acceptance.

- The manuscript addresses a highly relevant and contemporary topic in workplace coaching research.
- The objectives and rationale of the study are clearly stated and well justified.
- The study provides a comprehensive understanding of leadership coaching, executive coaching, and employee coaching.
- The literature review is detailed, systematic, and supported with relevant theoretical foundations.
- The use of PRISMA guidelines enhances the rigor and transparency of the review process.
- The methodology is clearly explained and sufficiently detailed for replication by future researchers.
- The use of Scopus and Web of Science databases strengthens the quality and reliability of the data sources.
- Bibliometric tools such as VOSviewer have been appropriately utilized for network and cluster analysis.
- The keyword co-occurrence analysis and thematic clustering are insightful and well interpreted.
- The identification of four thematic clusters significantly improves the understanding of workplace coaching literature.
- Tables, figures, and visualizations are informative, clear, and effectively support the findings.
- The discussion section is well connected with the bibliometric findings and existing literature.
- The conclusions are adequately supported by the results presented in the study.
- The manuscript provides useful practical implications for HR professionals, leaders, coaches, and organizations.
- The future research directions suggested are relevant, contemporary, and academically valuable.
- The manuscript demonstrates good academic writing quality and overall scholarly contribution.
- Overall, the study makes a meaningful contribution to workplace coaching research and is recommended for acceptance.

Are the rationale for, and objectives of, the Systematic Review clearly stated?

Yes

Is the statistical analysis and its interpretation appropriate?

Yes

If this is a Living Systematic Review, is the ‘living’ method appropriate and is the search schedule clearly defined and justified? (‘Living Systematic Review’ or a variation of this term should be included in the title.)

Yes

Are sufficient details of the methods and analysis provided to allow replication by others?

Yes

Are the conclusions drawn adequately supported by the results presented in the review?

Yes

Reviewer Expertise:

Marketing AnalyticsBusiness AnalyticsHuman Resource ManagementOrganizational BehaviourLeadership and Workplace DevelopmentResearch MethodologyBibliometric Analysis and Systematic Literature ReviewManagement and Business ResearchData Science Applications in ManagementOrganizational Psychology and Coaching Research

I confirm that I have read this submission and believe that I have an appropriate level of expertise to confirm that it is of an acceptable scientific standard.

# Reviewer report: Reviewer response for version 1

**DOI:** 10.5256/f1000research.189463.r447676

**Published:** 2026-01-13

**Authors:** Meenakshi Verma

**Affiliations:** 1 Symbiosis Centre for Management Studies, Bengaluru, India

**Summary:**

 The study identifies a significant growth in scholarly interest in workplace coaching, particularly post-2020, and delineates four dominant thematic clusters:

1. Coaching for workplace learning and development
2. Multifaceted advantages of executive and leadership coaching
3. Dimensions of workplace coaching effectiveness
4. Employee coaching for HRM and development

**Justifications:**

1. The rationale is clearly articulated. The authors convincingly argue that despite the growing popularity of workplace coaching, the literature remains fragmented, with limited comprehensive reviews integrating bibliometric techniques.
2. The methodology is generally well described. The authors specify databases, timeframe, inclusion and exclusion criteria, PRISMA stages, and software tools used.
3. This study does not conduct inferential statistical analysis or meta-analysis.
4. The main conclusions regarding publication growth, dominant themes, influential contributors, and conceptual evolution are well supported by the bibliometric findings.
5. The article does not claim to be a Living Systematic Review, nor does it define update cycles or continuous search protocols.

**Overall Strengths of the Manuscript:**

- Timely and relevant topic with strong interdisciplinary appeal
- Robust dataset (343 articles across 25 years)
- Effective integration of SLR and bibliometric methods

**Minor Required Revisions:**

 To ensure scientific soundness and methodological transparency, the following points must be addressed:

1. Provide full database-specific search strings
2. Report bibliometric software parameters
3. Align conclusions more strictly with bibliometric evidence
4. Clearly separate data-driven findings from future research propositions

Recommendation: Minor Revision

Are the rationale for, and objectives of, the Systematic Review clearly stated?

Yes

Is the statistical analysis and its interpretation appropriate?

Not applicable

If this is a Living Systematic Review, is the ‘living’ method appropriate and is the search schedule clearly defined and justified? (‘Living Systematic Review’ or a variation of this term should be included in the title.)

Not applicable

Are sufficient details of the methods and analysis provided to allow replication by others?

Partly

Are the conclusions drawn adequately supported by the results presented in the review?

Partly

Reviewer Expertise:

HR and OB

I confirm that I have read this submission and believe that I have an appropriate level of expertise to confirm that it is of an acceptable scientific standard.
